# ESBL-Producing *Klebsiella pneumoniae* in the Broiler Production Chain and the First Description of ST3128

**DOI:** 10.3389/fmicb.2018.02302

**Published:** 2018-10-03

**Authors:** Katrin Daehre, Michaela Projahn, Anika Friese, Torsten Semmler, Sebastian Guenther, Uwe H. Roesler

**Affiliations:** ^1^Institute for Animal Hygiene and Environmental Health, Freie Universität Berlin, Berlin, Germany; ^2^NG1-Microbial Genomics, Robert Koch Institute, Berlin, Germany; ^3^Pharmaceutical Biology Institute of Pharmacy, Ernst-Moritz-Arndt-Universität Greifswald, Greifswald, Germany

**Keywords:** extended-spectrum-beta-lactamases, ESBLs, *Klebsiella pneumoniae*, broiler production, broiler chicken

## Abstract

ESBL-producing *Klebsiella pneumoniae (K. pneumoniae)* represent an increasing problem both in human and veterinary medicine. As SHV-2 - encoding *K. pneumoniae* were recently detected in the broiler production we were interested in investigating a possible transmission along the broiler production chain and furthermore, in evaluating their possible impact on human health. Therefore, 41 ESBL-producing *K. pneumoniae* originating from a parent flock, from the hatcherys' environment during the hatching of that parent flocks' chickens, and from an associated fattening flock were investigated on an Illumina Miseq. Whole genome sequences were analyzed concerning their MLST-type, cgMLST-type, genotypic and phenotypic resistance, plasmid profiles and virulence genes. Irrespective of the origin of isolation all investigated isolates were multi-drug resistant, harbored the same ESBL-gene *bla*_SHV−2_, shared the same sequence type (ST3128) and displayed 100% similarity in core genome multilocus sequence typing (cgMLST). In addition, *in silico* plasmid typing found several Inc/Rep types associated with ESBL-plasmids. Summarizing, identical clones of SHV-2—producing *K. pneumoniae* were detected in different stages of the industrial broiler production in one out of seven investigated broiler chains. This proves the possibility of pseudo-vertical transmission of multi-resistant human pathogens from parent flocks to hatcheries and fattening flocks. Furthermore, the importance of cross-contamination along the production chain was shown. Although the ESBL-producing *K. pneumoniae* clone detected here in the broiler production has not been associated with clinical settings so far, our findings present a potential public health threat.

## Introduction

The emergence of extended-spectrum beta-lactamase-producing Enterobacteriaceae has been of particular interest for years, in both human and veterinary medicine. Especially *Klebsiella pneumoniae* (*K. pneumoniae)*, causing community and nosocomial infections of the respiratory and urinary tract as well as bloodstream infections are of critical concern. Resistance against antimicrobials leads to limited therapeutic options, resulting in increasing difficulties of treatments. In contrast to ESBL- producing *E. coli* which have become very common in veterinary medicine, especially in livestock, companion animals and wildlife, ESBL-producing *K. pneumoniae* were detected rarely in healthy broiler (Hiroi et al., 2012; Yossapol et al., 2017; Mahanti et al., 2018), diseased horses (Vo et al., 2007) dairy cows (Locatelli et al., 2010) and in companion animals (Ewers et al., 2014).

In general, the zoonotic impact of animal-originated pathogens on public health via direct contact or due to the consumption of contaminated meat is assumed (Smet et al., 2010; Marshall and Levy, 2011). This warrants the importance of investigations concerning multi-drug resistant bacteria in food-producing animals at different levels of production, to characterize the impact on humans. In previous studies, we detected ESBL-producing *K. pneumoniae* in a German hatchery (Projahn et al., 2017) as well as in a connected broiler farm (Daehre et al., 2017) in one out of seven investigated broiler chains. To characterize and compare those ESBL-producing *K. pneumoniae* strains detected at different levels of the broiler production we used whole genome sequencing assuring a high resolution of the clonal relationship. We are aiming at revealing possible transmission routes of ESBL-producing *K. pneumoniae* along the production pyramid as well as at assessing a possible impact on human health.

## Materials and methods

### Samples/flock

In the years 2014–2016, seven German parent flocks, their corresponding hatchlings in the hatchery and later on at the fattening farms as well as the environment of the hatchery and the respective farm were investigated for the occurrence of ESBL-/AmpC-producing Enterobacteriaceae as published by Projahn et al. (2017) and Daehre et al. (2017). There, in one fattening chain (chain B), SHV-2 -producing *K. pneumoniae* strains were detected and those stored bacterial isolates were retrospectively characterized in the present study. In detail, various samples were taken after the hatching of the chicken in the hatchery such as dust, air and swabs from the hatchery's environment. On the fattening farm individual animal samples as well as samples from the housing environment were collected at three different samplings (first day, middle and end of fattening period). In detail, 40 individual animals (cloacal swabs), pooled feces, boot swabs, litter, dust, and air were collected and several surfaces in the barn were swabbed (environmental swabs).

**Figure 1:**
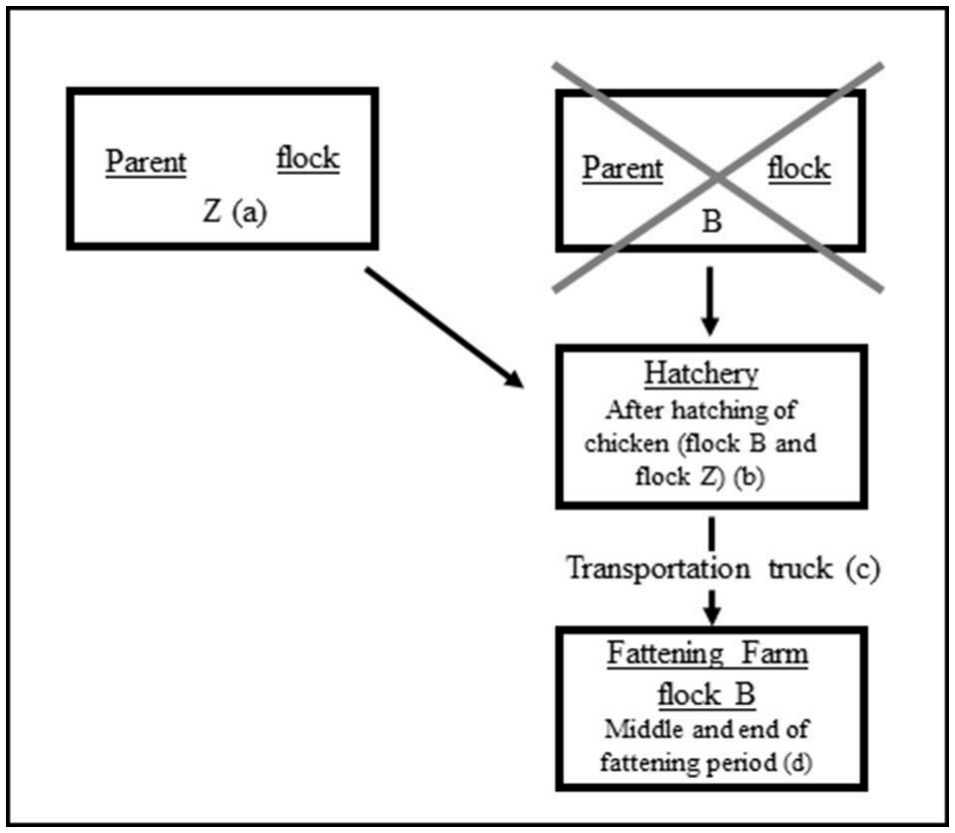
Schematic diagram of stages of the broiler production chain tested positive for SHV-2-producing *Klebsiella pneumoniae*. Parent flock B *was* negative. Positive samples: (a)-pooled feces; (b)-egg shells, environmental swab; (c)-swabs; (d)-cloacal swabs, pooled feces, boot swab, litter, dust, air, environmental swabs.

Within this study, additional isolates of a boot swab and a pooled feces sample from another parent flock (flock Z) were investigated as well. This was done due to the fact that the eggs of both parent flocks (B and Z) were bred in the hatchery at the same time. Furthermore, we analyzed isolates originating from environmental swabs from the truck that transported the chicken of parent flock B from the hatchery to the farm. Figure 1 schematically illustrates the origin of the *bla*_SHV−2_ - positive isolates. Detailed information on the investigated isolates are shown in Table 1 and in Table S1.

**Table 1:** Information on seven *K. pneumoniae* isolates (ST3128) including sampling timepoint, sample type, isolate ID, plasmid type, genotypic, and phenotypic resistance.

**Sampling timepoint**	**Sample type**	**Isolate ID**	**Plasmid type**	**Genotypic resistance**	**Phenotypic resistance**
I	PF	ITU10028	IncR, IncFIB, IncFII, IncHI1B, Col	*bla*_SHV−2_, *bla*_SHV−1_, *fos*A-like, *sul*1*, dfr*A12*, tet*(D)*, aad*A1*, aad*A2*, aac*(3)-like, oqxA-like, *oqx*B-like, *parC* S80I mutation	
II	EggS	ITU10022	IncR, IncFIB, IncFII, IncHI1B, IncX1, IncX3, Col	*bla*_SHV−2_, *bla*_SHV−1_, *fos*A-like, *sul*1*, dfr*A12*, tet*(D)*, aad*A1*, aad*A2, *aac(3)-like*, oqxA-like, *oqx*B-like, *parC* S80I mutation	
III	EnvS	ITU10024,	IncR, IncFIB, IncFII, IncHI1B, Col	*bla*_SHV−2_, *bla*_SHV−1_, *fos*A-like, *sul*1, *dfr*A12, *tet*(D), *aad*A1, *aad*A2, *aac*(3)-like, *oqxA-like, oqxB-like, parC* S80I mutation	AMX, AMC, AMP, SAM, CFR, CFL, CLT, CFZ,
IV	CS	ITU3949	IncR, IncFIB, IncFII, Col	*bla*_SHV−2_, *bla*_SHV−1_, *fos*A-like, *sul*1*, dfr*A12, *tet*(D), *aad*A1, *aad*A2, *aac*(3)-like, *oqx*A-like, *oqx*B-like, *parC* S80I mutation	CFM, CPZ, CTX, CFV, CFP, CPD, CAZ, CEX,
	Litter	ITU3854	IncR, IncFIB, IncFII, IncHI1B, Col	*bla*_SHV−2_, *bla*_SHV−1_, *fos*A-like, *sul*1, *dfr*A12, *tet*(D), *aad*A1, *aad*A2, *aac*(3)-like, *oqxA*-like, *oqxB*-like, *parC* S80I mutation	CFX, DOX, ENR, GEN, MAR, PIP, PUFX, TET, TOB, SXT
V	CS 18	ITU4179	IncR, IncFIB, IncFII, IncHI1B, Col	*bla*_SHV−2_, *bla*_SHV−1_, *fos*A-like, *sul*1, *dfr*A12, *tet*(D), *aad*A1, *aad*A2, *aac*(3)-like, *oqx*A-like, *oqx*B-like, *Qnr*S1-like, *parC* S80I mutation	
	BS	ITU4097	IncR, IncFIB, IncFII, IncHI1B, Col	*bla*_SHV−2_, *bla*_SHV−1_, *fos*A-like, *sul*1, *dfr*A12, *tet*(D), *aad*A1, *aad*A2, *aac*(3)-like, *oqx*A-like, *oqx*B-like, *parC* S80I mutation	

*I, parent flock Z; II, hatchery, after hatching; III, truck (from hatchery to farm); IV, fattening farm, middle of fattening period; V, fattening farm, end of fattening period; PF, pooled feces; EggS, pooled eggshells; EnvS, environmental swab; CS, cloacal swab, BS, boot swab; ITU, Institut für Tier- und Umwelthygiene; AMX, amoxicillin; AMC, Amoxicillin/clavulanic acid; AMP, ampicillin; SAM, ampicillin/sulbactam; CFR, cefadroxil; CFL, cefalexin; CLT, cefalotin; CFZ, cefazolin; CFM, cefixime; CPZ, cefoperazone; CTX, cefotaxime; CFV, cefovecin; CFP, cefpirome; CPD, cefpodoxime; CAZ, ceftazidime; CEX, ceftiofur; CFX, cefuroxime; DOX, doxycyclin; ENR, enrofloxacin; GEN, gentamicin; MAR, marbofloxacin; PIP, piperacillin; PUFX, prulifloxacin; TET, tetracyclin; TOB, tobramycin; SXT, trimethoprim/sulfamehoxazol*.

### Laboratory methods

All samples were processed as described by Projahn et al. (2017) and Daehre et al. (2017). Finally, the samples were streaked out on MacConkey No. 3 (Oxoid, Wesel, Germany) agar plates with the addition of 1 mg/l cefotaxime (AppliChem, Darmstadt, Germany). The species of all isolates with Enterobacteriaceae-like phenotypes were determined by MALDI-TOF analyses. The detection of the most common class A beta-lactamase-genes including *bla*_SHV_ was performed as described by Roschanski et al. (2014) and verified by sequencing using the same primer set as published by Projahn et al. (2017).

Forty-one *K. pneumoniae* isolates with *bla*_SHV−2_ - genes from 26 different samples (up to three isolates per sample) were chosen for further characterization. These isolates originated from the other parent flock Z, the hatchery, the truck (transport of chicken from hatchery to farm) as well as from samples from the middle and the end of the fattening period on the farm (cloacal swabs and samples from the housing environment; Figure 1, Table 1, and Table S1).

The Vitek®2 system (BioMérieux, Germany; card GN38) was used to determine phenotypic antimicrobial resistance to various ß-lactam-antibiotics and other classes of antimicrobials.

To get more information concerning the phylogenetic relationship of the different samples, whole genome sequencing (WGS) was performed. Therefore, DNA was extracted with the MasterPure™ DNA purification kit (Epicenter, Illumina) and Illumina MiSeq 300-bp paired-end with a coverage between 50x and 120x was used. Following a quality control performed with the NGS tool kit (Patel and Jain, 2012) high quality reads were defined (minimum of 70% of bases having a phred score higher than 20) and *de novo* assembled into contiguous sequences (contigs) using CLC Genomics workbench 9.0 (Qiagen, Venlo, Netherlands). These sequence data have been deposited at DDBJ/ENA/GenBank and the accession numbers can be found in the Table S2.

The *bla*_SHV_ genes were sequenced using the same primer set as published by Projahn et al. (2017) and evaluated with BioNumerics 6.6.

WGS data were used for genotypic characterization utilizing the Center for Genomic Epidemiology (Center for Genomic Epidemiology, 2018): multi-locus sequence types [MLST; MLSTFinder 1.8 (Larsen et al., 2012)], plasmids [PlasmidFinder 1.3 (Carattoli et al., 2014)] and resistance genes [ResFinder3.0 (Zankari et al., 2012)] were determined. Additionally, core genome MLST (cgMLST) typing was performed using the cgMLST.org Nomenclature Server^1^ and Ridom Seqsphere 4.1 (Ridom GmbH, Muenster, Germany). Within cgMLST, for *K. pneumoniae*, 2358 conserved genome-wide genes are compared, resulting in a very high discriminatory power.

BLAST analyses of the assembled contigs were done and the accordance of *bla*_SHV_-carrying contigs with SHV-encoding plasmids described for *K. pneumoniae* (JX461340.1, CP025463, CP025458, DQ449578, JN247852, and others) were checked using the European nucleotide archive^2^, the European Center for Biotechnology Information^3^ and features of Geneious v. 7.1.2 (Kearse et al., 2012) and DNASTAR® Lasergene 11 SeqMan Pro™ (version 11.2.1). The genetic vicinity of the bla*SHV* region as well as the occurrence of genes known for the association with virulent *K. pneumoniae* (*magA, rmpA, entB, iutA, YbtS, Kfu, allS, mrkD, wzi*; Compain et al., 2014) were investigated using the same tools.

## Results and discussion

SHV-2 -encoding *K. pneumoniae* strains were detected in all investigated stages of the broiler production chain: 25% of the samples from parent flock Z (1/4) (but not in samples from parent flock B), 22.2% from the hatchery's environment (2/9) and 28.8% of individual animals (23/80) resp. 68.2% of samples from the housing environment (15/22) of flock B (middle and end of fattening period) were tested positive. All 41 *K. pneumoniae* isolates were multi-drug resistant, harbored the genes for the ESBL beta-lactamase SHV-2 and were assigned to the newly described MLST type ST3128. cgMLST typing revealed 100% similarity. Therefore, a clonal relationship between the isolates detected at the different stages of the broiler production chain can be stated.

Antibiotic resistant *K. pneumoniae* isolates, especially ESBL–and/or carbapenemase–producers with resistance toward third/fourth generation cephalosporins and carbapenems are of great concern in both human and veterinary medicine. To our knowledge, this is the first finding of ESBL-producing *K. pneumoniae* detected in various stages within the same broiler production chain. To get more information on these strains, 41 isolates detected in samples from a parent flock, the hatchery, the transportation vehicle and a fattening flock of one out of seven investigated broiler chains were further characterized.

All *K. pneumoniae* isolates harbor the *bla*_SHV−2_ - gene, belong to the previously unknown *K. pneumoniae* MLST type ST3128 and show phenotypical resistance against various antimicrobials including third and fourth generation cephalosporins, but not against carbapenems (Table 1 and Table S1). With regard to the fluoroquinolone resistance, all 41 isolates have a mutation (S80I) in the *parC* gene known for fluoroquinolone-resistant *K. pneumoniae* (Correia et al., 2017) but mutations in the QRDR-region of the *gyrA* gene were not detected. All investigated *K. pneumoniae* isolates possess *entB* and *mrkD*, but none of the other genes, associated with virulence of *K. pneumoniae* (*magA, rmpA, iutA, YbtS, Kfu, allS, wzi)*. Enterobactin *(entB)* is a prototypical catecholate siderophore as part of iron acquisition systems. But *entB* only is known for virulence when occurring in a combination with iron acquisition systems (*iutA, YbtS, Kfu*). The same applies to *mrkD*. *mrkD* is believed to function as the type 3 fimbrial adhesion and mediates binding to extracellular matrix (Jagnow and Clegg, 2003) but in virulent *K. pneumoniae* strains only occurs in combination with other virulence factors, which were tested negative in our isolates. Therefore, the 41 ESBL-producing *K. pneumoniae* strains, detected in healthy broiler chicken, do not harbor virulence genes that were described in any clinical association. Inc typing using plasmidFinder found the plasmid types IncR, IncFIB, IncFII, and IncHI1B known for the presence of ESBL-encoding genes in all strains (Table 1 and Table S1). The DNA sequences of the *bla*_SHV−2_ carrying contigs of ~7,400 bp length were homologous to plasmid p1658/97 from *Eschericia coli* (accession number: AF550679) and plasmid pSEM from *Salmonella enterica* (AJ245670), containing a *recF* gene upstream and a *deoR* gene downstream from the *bla*_SHV−2_. These genes are also present in a *bla*_SHV_-carrying plasmid of *K. pneumoniae* published by Yu et al. (2006). The *recF* gene may contribute to the mobilization of the *bla* gene to a plasmid via the *recF* recombination pathway (Kolodner et al., 1985).

Additionally, a bla-SHV-2 carrying fragment (~3,500 bp) of the contigs was detected in plasmids of *K. pneumoniae* (JX461340.1, CP025463, CP025458, DQ449578, JN247852) (more than 99% identity). The adjacent DNA sequence was identical to other plasmids (CP027613, LT985275, and others). These findings make the location of the *bla*_SHV−2_ on a plasmid very likely.

To elucidate the epidemiological relationship and, therefore, the transmission dynamics along the production chain, cgMLST was performed. cgMLST, comparing 2358 genes for *K. pneumoniae*, revealed 100% similarity. This demonstrates that identical clones of *K. pneumoniae* (ST3128), encoding for SHV-2 were detected in the different stages of the broiler production chain pointing toward ongoing transmission processes.

The circulation of ESBL-/AmpC-producing Enterobacteriaceae along the broiler production process was described in previous studies (Dierikx et al., 2013; Nilsson et al., 2014). Projahn et al. (2017) conducted transmission investigations with a special focus on the hatchery and *Escherichia coli (E. coli)*. There, the introduction of ESBL-producing *E. coli* strains directly from the parent flock into the hatchery, despite the eggs' disinfection, was shown. Additionally, a pseudo-vertical transmission, in detail, the introduction of ESBL-producing Enterobacteriaceae into the hatchery and the chickens' colonization by the uptake of resistant bacteria from the environment of the hatchery was discussed.

Our results reinforce these hypotheses. Parent flock Z (the other parent flock) was tested positive for SHV-2–producing *K. pneumoniae*. In the hatchery, clones of this strain were detected after the chickens hatching in the hatchery's environment (environmental swab and eggshells). We showed that the chicken of flock B, that hatched at the same time in the hatchery were colonized with those clones. Furthermore, the SHV-2-producing *K. pneumoniae* were detected during the whole fattening period of flock B both, in individual animals as well as in samples from the environment. This clearly demonstrates a transmission from parent flocks, via the hatchery, into the fattening flocks. This is in accordance to Projahn et al. (2018). They showed that ESBL-producing *E. coli* were already present in the hatchery and colonized the recently hatched chickens. Therefore, they also confirmed the hatchery as a contamination source for the fattening period.

In our study, clones of the SHV-2-producing *K. pneumoniae* were detected in parent flock Z, in the hatchery after the chickens hatching and during the fattening period of flock B. As the eggs of parent flock Z were bred at the same time as the eggs of flock B, the chicken of flock Z and B hatched at the same time in the same surrounding. This means the original source of the ESBL-producing *K. pneumoniae* strains was not the respective parent flock B but an unrelated parent flock. This clearly emphasizes the importance of cross-contamination via the environment, especially at hatchery-level.

As described by Dierikx et al. (2013) the broiler production system seems rather simple: only a few breeding companies produce broilers for many farms. The breeding eggs of several parent flocks are processed in a few hatcheries and the hatched broiler chicken are delivered to various fattening farms. Thus, the introduction of resistant bacteria from one parent flock into the hatchery can cause the spread of these strains in several fattening farms. Consequently, the absence of antibiotic resistant bacteria in parent flocks is essential to minimize the occurrence of these bacteria in the production pyramid and one high prevalent parent flock has the potential to contaminate all hatchlings of one course of hatching.

Besides, *K. pneumoniae* clones were also detected in the truck, transporting the chicken from the hatchery to the farms (environmental swabs from the truck's ground and walls). Projahn et al. (2018) also detected ESBL- producing Enterobacteriaceae in a transportation truck and indicated a transmission of resistant bacteria via the transportation process. As the trucks transport animals for several farms, cross-contamination even during the transport could be of importance as well.

As described above, the *K. pneumoniae* isolates were detected during the investigation on ESBL-/AmpC-producing Enterobacteriaceae in parent flocks, the hatchery and at fattening farms (Daehre et al., 2017; Projahn et al., 2017). Thus, next to the *K. pneumoniae*, several ESBL-/AmpC- producing *E. coli* were detected. These *E. coli* isolates encoded for SHV-12, CMY-2 or CTX-M-1, but not for SHV-2, in contrast to the *K. pneumoniae* isolates. Therefore, with our investigated isolates we cannot show any association between ESBL- producing *E. coli* and ESBL-producing *K. pneumoniae* in the broiler production chain.

The detected antibiotic resistant *K. pneumoniae* strains of ST3128 have not been reported in clinical settings, yet, and, therefore, did not have an impact on human health so far. However, plasmids with resistance genes are transferable between strains and species. Therefore, resistance-carrying plasmids detected in food-producing animals always pose a possible risk for human health.

In summary, our results demonstrate the presence of SHV-2–producing *K. pneumoniae* clones in several stages of the broiler production pyramid. A pseudo-vertical transmission of ESBL-producing *K. pneumoniae*, resulting in a positive fattening flock caused by the uptake of bacteria that were introduced into the hatchery by another parent flock was shown for the first time. This also indicates the importance of cross-contamination. As *K. pneumoniae* of ST3128 have not been known previously, clones of our strains have never been reported in clinical associations. Therefore, to date, these strains did not have an impact on human health. Nevertheless, a reduction of antibiotic-resistant bacteria in food-producing animals should be achieved, in order to not worsen the situation in human and veterinary medicine.

## Author contributions

UR and AF designed the study. KD and MP performed the samplings and the laboratory work. TS and SG performed bioinformatic analysis. KD analyzed the data and wrote the manuscript. All authors have read and approved the final draft of the article.

### Conflict of interest statement

The authors declare that the research was conducted in the absence of any commercial or financial relationships that could be construed as a potential conflict of interest.

## Footnotes

^1^“cgMLST.org Nomenclature Server,”. Available online at: http://www.cgmlst.org/ncs (Accessed April 05, 2018).

^2^European Nucleotide Archive. Available online at: https://www.ebi.ac.uk/ena (Accessed April 05, 2018).

^3^“National Center for Biotechnology Information,”. Available online at: https://www.ncbi.nlm.nih.gov/ (Accessed April 05, 2018).
