# Supplementary material for: ESBL-Producing Klebsiella pneumoniae in the Broiler Production Chain and the First Description of ST3128
Source: Front Microbiol. 2018 Oct 3;9:2302. doi: 10.3389/fmicb.2018.02302 (PMC6178893; doi:10.3389/fmicb.2018.02302)
Supplement: Supplementary file 1 [file Table_1.docx]

Table S1: Information on 41 K. pneumoniae isolates (ST3128) including sampling timepoint, sample type, isolate ID, plasmid type, genotypic and phenotypic resistance.

|  |  |  |  |  |  |  |
| --- | --- | --- | --- | --- | --- | --- |
| **Sampling**  **timepoint** | **Sample**  **type** |  | **Isolate ID** | **Plasmid type** | **Genotypic resistance** | **Phenotypic resistance** |
| I | PF |  | ITU10032 | IncR, IncFIB, IncFII, IncHI1B, IncX3, Col | *bla*_SHV-2,_, *bla*_SHV-1_, fosA-like, sul1, dfrA12, tet(D), aadA1, aadA2,aac(3)-like, oqxA-like, oqxB-like, QnrS1, *parC* S80I mutation | AMX, AMC, AMP, SAM, CFR, CFL, CLT, CFZ, CFM, CPZ, CTX, CFV, CFP, CPD, CAZ, CEX, CFX, DOX, ENR, GEN, MAR, PIP, PUFX, TET, TOB, SXT |
|  |  |  | ITU10028 | IncR, IncFIB, IncFII, IncHI1B, Col | *bla*_SHV-2_, fosA-like, sul1, dfrA12, tet(D), aadA1, aadA2,aac(3)-like, oqxA-like, oqxB-like, *parC* S80I mutation |  |
|  |  |  |  |  |  |  |
| II | EggS |  | ITU10022 | IncR, IncFIB, IncFII, IncHI1B, IncX1, IncX3, Col | *bla*_SHV-2,_, *bla*_SHV-1_,fosA-like, sul1, dfrA12, tet(D), aadA1, aadA2,aac(3)-like, oqxA-like, oqxB-like, *parC* S80I mutation |  |
|  | EnvSw^a^ |  | ITU10030 | IncR, IncFIB, IncFII, IncHI1B, Col | *bla*_SHV-2,_, *bla*_SHV-1_, fosA-like, sul1, dfrA12, tet(D), aadA1, aadA2,aac(3)-like, oqxA-like, oqxB-like, QnrS1, *parC* S80I mutation |  |
|  |  |  |  |  |  |  |
| III | Truck |  | ITU10024, | IncR, IncFIB, IncFII, IncHI1B, Col | *bla*_SHV-2,_, *bla*_SHV-1_,fosA-like, sul1, dfrA12, tet(D), aadA1, aadA2,aac(3)-like, oqxA-like, oqxB-like, *parC* S80I mutation |  |
|  |  |  | ITU10031 | IncR, IncFIB, IncFII, IncHI1B, Col | *bla*_SHV-2,_,  *bla*_SHV-1_, fosA-like, sul1, dfrA12, tet(D), aadA1, aadA2,aac(3)-like, oqxA-like, oqxB-like, *parC* S80I mutation |  |
|  |  |  | ITU10025 | IncR, IncFIB, IncFII, Col | *bla*_SHV-2,_, *bla*_SHV-1_, fosA-like, sul1, dfrA12, tet(D), aadA1, aadA2,aac(3)-like, oqxA-like, oqxB-like, *parC* S80I mutation |  |
|  |  |  |  |  |  |  |
| IV | CS 16 |  | ITU3949 | IncR, IncFIB, IncFII, Col | *bla*_SHV-2,_, *bla*_SHV-1_, fosA-like, sul1, dfrA12, tet(D), aadA1, aadA2,aac(3)-like, oqxA-like, oqxB-like, *parC* S80I mutation |  |
|  |  |  | ITU3951 | IncR, IncFIB, IncFII, IncHI1B, Col | *bla*_SHV-2,_, *bla*_SHV-1_, fosA-like, sul1, dfrA12, tet(D), aadA1, aadA2,aac(3)-like, oqxA-like, oqxB-like, *parC* S80I mutation |  |
|  |  |  | ITU4064 | IncR, IncFIB, IncHI1B, Col | *bla*_SHV-2,_, *bla*_SHV-1_, fosA-like, sul1, dfrA12, tet(D), aadA1, aadA2,aac(3)-like, oqxA-like, oqxB-like, *parC* S80I mutation |  |
|  |  |  |  |  |  |  |
|  | CS 25 |  | ITU3971, ITU3978 | IncR, IncFIB, IncFII, IncHI1B, Col | *bla*_SHV-2,_, *bla*_SHV-1_, fosA-like, sul1, dfrA12, tet(D), aadA1, aadA2,aac(3)-like, oqxA-like, oqxB-like, *parC* S80I mutation |  |
|  |  |  | ITU3972 | IncR, IncFIB, IncFII, IncHI1B, Col | *bla*_SHV-2,_, *bla*_SHV-1_, fosA-like, sul1, dfrA12, tet(D), aadA1, aadA2,aac(3)-like, oqxA-like, oqxB-like, QnrS1-like, *parC* S80I mutation |  |
|  |  |  |  |  |  |  |
|  | CL 31 |  | ITU3985, ITU3986, ITU3987 | IncR, IncFIB, IncFII, IncHI1B, Col | *bla*_SHV-2,_, *bla*_SHV-1_, fosA-like, sul1, dfrA12, tet(D), aadA1, aadA2,aac(3)-like, oqxA-like, oqxB-like, *parC* S80I mutation |  |
|  |  |  |  |  |  |  |
|  | EnvSw^b^ |  | ITU3908 | IncR, IncFIB, IncFII, IncHI1B, Col | *bla*_SHV-2,_, *bla*_SHV-1_, fosA-like, sul1, dfrA12, tet(D), aadA1, aadA2,aac(3)-like, oqxA-like, oqxB-like, *parC* S80I mutation |  |
|  | EnvSw^c^ |  | ITU3914 | IncR, IncFIB, IncFII, IncHI1B, Col | *bla*_SHV-2,_, *bla*_SHV-1_, fosA-like, sul1, dfrA12, tet(D), aadA1, aadA2,aac(3)-like, oqxA-like, oqxB-like, *parC* S80I mutation |  |
|  | EnvSw^d^ |  | ITU3917 | IncR, IncFIB, IncFII, IncHI1B, Col | *bla*_SHV-2,_, *bla*_SHV-1_, fosA-like, sul1, dfrA12, tet(D), aadA1, aadA2,aac(3)-like, oqxA-like, oqxB-like, *parC* S80I mutation |  |
|  | Air |  | ITU3896 | IncR, IncFIB, IncFII, IncHI1B, Col | *bla*_SHV-2,_, *bla*_SHV-1_, fosA-like, sul1, dfrA12, tet(D), aadA1, aadA2,aac(3)-like, oqxA-like, oqxB-like, *parC* S80I mutation |  |
|  |  |  | ITU3897 | IncR, IncFIB, IncFII, IncHI1B, Col | *bla*_SHV-2,_, *bla*_SHV-1_, fosA-like, sul1, dfrA12, tet(D), aadA1, aadA2,aac(3)-like, oqxA-like, oqxB-like, *parC* S80I mutation |  |
|  | Dust |  | ITU3887 | IncR, IncFIB, IncFII, IncHI1B, IncQI, Col | *bla*_SHV-2,_, *bla*_SHV-1_, fosA-like, sul1, dfrA12, tet(D), aadA1, aadA2,aac(3)-like, oqxA-like, oqxB-like, *parC* S80I mutation |  |
|  | PF |  | ITU3881 | IncR, IncFIB, IncFII, IncHI1B, Col | *bla*_SHV-2,_, *bla*_SHV-1_, fosA-like, sul1, dfrA12, tet(D), aadA1, aadA2,aac(3)-like, oqxA-like, oqxB-like, *parC* S80I mutation |  |
|  | BS |  | ITU3868, ITU3872 | IncR, IncFIB, IncFII, IncHI1B, Col | *bla*_SHV-2,_, *bla*_SHV-1_, fosA-like, sul1, dfrA12, tet(D), aadA1, aadA2,aac(3)-like, oqxA-like, oqxB-like, *parC* S80I mutation |  |
|  | Feed |  | ITU3862 | IncR, IncFIB, IncFII, IncHI1B, Col | *bla*_SHV-2,_, *bla*_SHV-1_, fosA-like, sul1, dfrA12, tet(D), aadA1, aadA2,aac(3)-like, oqxA-like, oqxB-like, *parC* S80I mutation |  |
|  | Litter |  | ITU3854 | IncR, IncFIB, IncFII, IncHI1B, Col | *bla*_SHV-2,_, *bla*_SHV-1_, fosA-like, sul1, dfrA12, tet(D), aadA1, aadA2,aac(3)-like, oqxA-like, oqxB-like, *parC* S80I mutation |  |
| V | CS 18 |  | ITU4179, ITU4180, ITU4181 | IncR, IncFIB, IncFII, IncHI1B, Col | *bla*_SHV-2,_, *bla*_SHV-1_, fosA-like, sul1, dfrA12, tet(D), aadA1, aadA2,aac(3)-like, oqxA-like, oqxB-like, QnrS1-like, *parC* S80I mutation |  |
|  |  |  |  |  |  |  |
|  | CS 30 |  | ITU4954 | IncR, IncFIB, IncFII, IncHI1B, Col | *bla*_SHV-2,_, *bla*_SHV-1_, fosA-like, sul1, dfrA12, tet(D), aadA1, aadA2,aac(3)-like, oqxA-like, oqxB-like, *parC* S80I mutation |  |
|  |  |  | ITU4956 | IncR, IncFIB, IncFII, IncHI1B, Col | *bla*_SHV-2,_, *bla*_SHV-1_, fosA-like, sul1, dfrA12, tet(D), aadA1, aadA2,aac(3)-like, oqxA-like, oqxB-like, *parC* S80I mutation |  |
|  |  |  | ITU4955 | IncR, IncFIB, IncFII, IncX3, Col | *bla*_SHV-2,_, *bla*_SHV-1_, fosA-like, sul1, dfrA12, tet(D), aadA1, aadA2,aac(3)-like, oqxA-like, oqxB-like, QnrS1-like, *parC* S80I mutation |  |
|  |  |  |  |  |  |  |
|  | SC 32 |  | ITU4963, ITU4965 | IncR, IncFIB, IncFII, IncHI1B, Col | *bla*_SHV-2,_, *bla*_SHV-1_, fosA-like, sul1, dfrA12, tet(D), aadA1, aadA2,aac(3)-like, oqxA-like, oqxB-like, *parC* S80I mutation |  |
|  |  |  | ITU4962 | IncR, IncFIB, IncFII, IncHI1B, Col | *bla*_SHV-2,_, *bla*_SHV-1_, fosA-like, sul1, dfrA12, tet(D), aadA1, aadA2,aac(3)-like, oqxA-like, oqxB-like, QnrS1, *parC* S80I mutation |  |
|  |  |  |  |  |  |  |
|  | Litter |  | ITU4077 | IncR, IncFIB, IncFII, Col | *bla*_SHV-2,_, *bla*_SHV-1_, fosA-like, sul1, dfrA12, tet(D), aadA1, aadA2,aac(3)-like, oqxA-like, oqxB-like, *parC* S80I mutation |  |
|  | BS |  | ITU4097 | IncR, IncFIB, IncFII, IncHI1B, Col | *bla*_SHV-2,_, *bla*_SHV-1_, fosA-like, sul1, dfrA12, tet(D), aadA1, aadA2,aac(3)-like, oqxA-like, oqxB-like, *parC* S80I mutation |  |
|  | PF |  | ITU4115 | IncR, IncFIB, IncHI1B, Col | *bla*_SHV-2,_, *bla*_SHV-1_, fosA-like, sul1, dfrA12, tet(D), aadA1, aadA2,aac(3)-like, oqxA-like, oqxB-like, *parC* S80I mutation |  |
|  | Dust |  | ITU4128 | IncR, IncFIB, IncFII, IncHI1B, Col | *bla*_SHV-2,_, *bla*_SHV-1_, fosA-like, sul1, dfrA12, tet(D), aadA1, aadA2,aac(3)-like, oqxA-like, oqxB-like, *parC* S80I mutation |  |
|  | EnvSw^e^ |  | ITU4141 | IncR, IncFIB, IncFII, IncHI1B, Col | *bla*_SHV-2,_, *bla*_SHV-1_, fosA-like, sul1, dfrA12, tet(D), aadA1, aadA2,aac(3)-like, oqxA-like, oqxB-like, *parC* S80I mutation |  |
|  |  |  |  |  |  |  |

I -parent flock Z; II – hatchery, after hatching; III – truck (from hatchery to farm); IV – fattening farm, middle of fattening period; V – fattening farm, end of

fattening period;

PF – pooled feces; EggS – pooled eggshells; EnvSw – environmental swab; CS – cloacal swab, BS – boot swab

^a^ - conveyor; ^b^ - feeding trough; ^c^ - wall; ^d^ ventilator; ^e^ - hangers of barns equipment

ITU – Institut für Tier- und Umwelthygiene; AMX – amoxicillin; AMC - Amoxicillin/clavulanic acid; AMP – ampicillin; SAM – ampicillin/sulbactam; CFR – cefadroxil; CFL – cefalexin; CLT – cefalotin; CFZ – cefazolin; CFM – cefixime; CPZ – cefoperazone; CTX – cefotaxime; CFV – cefovecin; CFP – cefpirome; CPD – cefpodoxime; CAZ – ceftazidime; CEX – ceftiofur; CFX – cefuroxime; DOX – doxycyclin; ENR – enrofloxacin; GEN – gentamicin; MAR – marbofloxacin; PIP – piperacillin; PUFX – prulifloxacin; TET – tetracyclin; TOB – tobramycin; SXT - trimethoprim/sulfamehoxazol
